# A case of oro-facial digital syndrome

**DOI:** 10.11604/pamj.2022.43.151.34814

**Published:** 2022-11-22

**Authors:** Ayushi Shashikant Gurharikar, Gagandeep Lamba

**Affiliations:** 1 Department of Pediatric and Preventive Dentistry, VSPM's Dental College and Research Center, Nagpur, Maharashtra, India

**Keywords:** Orofacial digital syndrome, OFD1 gene mutation, pediatric dentistry

## Image in medicine

An 8-year-old female patient was brought to consultation for poor aesthetics. On clinical examination, oral features included several over-retained carious deciduous teeth and congenitally missing permanent teeth. Extra-oral examination revealed various facial and digital deformities. Facial deformities involved hypertelorism along with flat nasal bridge, strabismus, or crossed eyes - a condition in which the eyes do not line up with one another, incompetent lips and retrognathic mandible. Digital deformities included syndactyly (a congenital condition characterized by the fusion of the bone or skin in the foot digits) and oligodactyly (presence of fewer than five toes).

**Figure 1:**
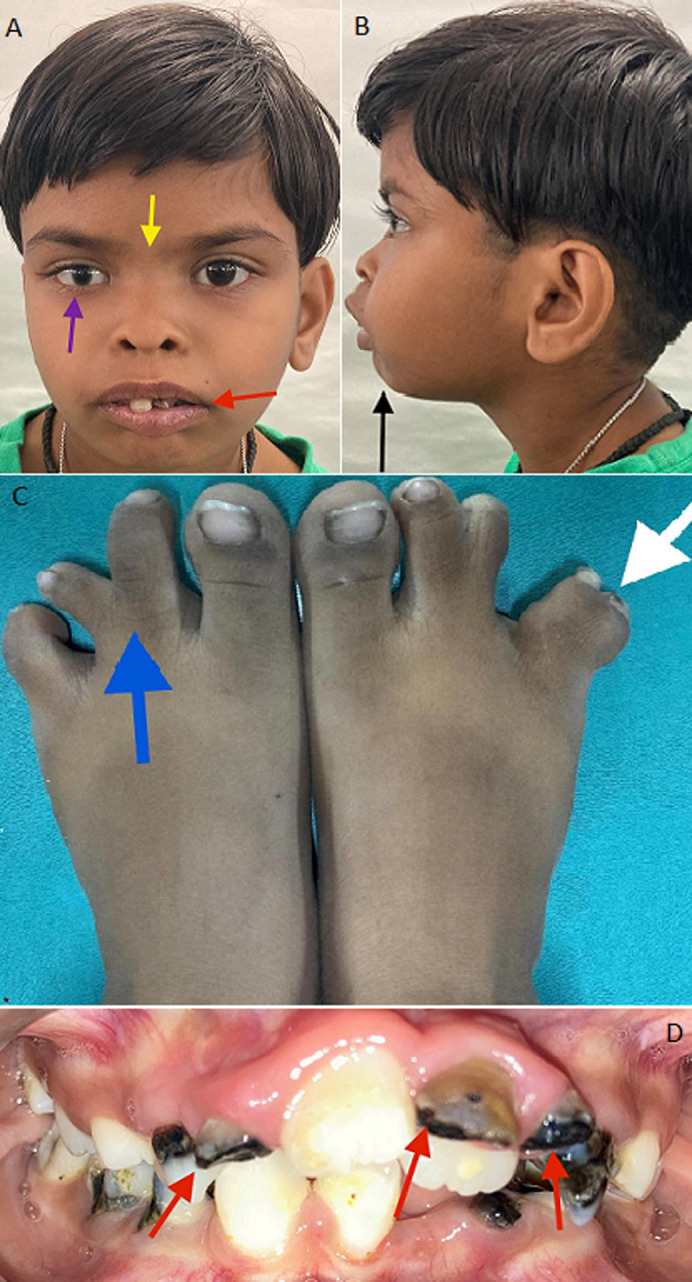
A) hypertelorism with a flat nasal bridge (yellow arrow) - strabismus or crossed eye (purple arrow) - incompetent lips (red arrow); B) retrognathic mandible; C) white arrow showing syndactyly and blue arrow showing oligodactyly; D) red arrows showing multiple over-retained teeth

